# ​Treadmill running modulates infrapatellar fat pad remodeling in osteoarthritic rats in an intensity-dependent manner

**DOI:** 10.1186/s13102-026-01561-8

**Published:** 2026-02-05

**Authors:** Zhi Chen, Gege Li, Haimei Deng, Zihang Hu, Qingwei Wang, Zijun He

**Affiliations:** 1 Department of Rehabilitation Medicine, The Tenth Affiliated Hospital, Southern Medical University (Dongguan people’s hospital), Wanjiang District 510280, Dongguan, Guangdong China; 2 Department of Rehabilitation,Guangdong Provincial Hospital of Chinese Medicine , The Second Affiliated Hospital of Guangzhou University of Chinese Medicine, Guangzhou, China; 3 Dongguan Key Specialty of Traditional Chinese Medicine (Rehabilitation Department), Dongguan, China; 4 Dongguan Experimental Centre for Sports Rehabilitation Research, Dongguan, China

**Keywords:** Osteoarthritis, Infrapatellar fat pad, Treadmill running, Fibrosis, Inflammation

## Abstract

**Objective:**

This study aimed to investigate the influence of treadmill running at varying intensities on the remodeling of the infrapatellar fat pad (IFP) in a rat model of osteoarthritis (OA), and to explore the association between exercise-induced IFP alterations and joint pain and structural integrity.

**Methods:**

Thirty male Wistar rats were randomly divided into five groups: control, OA, and three OA groups subjected to treadmill running intensities at low (OAL), medium (OAM), and high (OAH). OA was induced via intra-articular injection of monoiodoacetate. Following induction, rats in the running groups completed a 4-week treadmill running program. Comprehensive evaluations included histomorphological and immunohistochemical analyses, micro-computed tomography, and weight-bearing asymmetry tests to assess pain. Lastly, correlation analyses were performed to assess the relationship between IFP remodeling and joint pain or structural integrity.

**Results:**

The OAM group exhibited significant improvements in IFP remodeling, characterized by reduced fibrosis and inflammation (*p* < 0.05), accompanied by decreased M1 macrophage polarization. Cartilage integrity was better preserved (*p* < 0.05), and subchondral cortical bone mineral density was significantly higher (*p* < 0.01) in this group. Likewise, pain severity was significantly alleviated in the OAM group (*p* < 0.05). In contrast, the OAL and OAH groups displayed no significant improvements. Finally, correlation analysis demonstrated significant associations between IFP fibrosis/inflammation and cartilage damage (*r* = 0.60), pain (*r* = 0.58), and trabecular separation (*r* = 0.38).

**Conclusion:**

Treadmill running exerts intensity-dependent effects on IFP remodeling in OA rats, with moderate intensity providing optimal benefits. The significant correlations observed between IFP remodeling and joint integrity and pain indicate a potential link between IFP and exercise-induced joint protection.

## Introduction

Osteoarthritis (OA), traditionally considered a cartilage-centric disorder, is currently recognized as a whole-joint syndrome encompassing articular cartilage degradation, synovial inflammation, subchondral bone sclerosis, and infrapatellar fat pad (IFP) remodeling [1]. The IFP is a mass of adipose tissue located within the knee joint cavity, below the patellar tendon and adjacent to the synovium. Under physiological conditions, it functions as a cushion, dissipating mechanical stress and protecting the knee joint from excessive load [2]. Furthermore, it assists in maintaining joint stability by stabilizing the patella at the extremes of knee motion [3]. However, in the pathological environment of OA, the IFP undergoes remodeling, characterized by fibrosis and inflammation [4]. IFP fibrosis renders the tissue heterogeneous, stiffer, and less compliant, resulting in aberrant mechanical stress distribution on the cartilage and ultimately leading to its degradation [5]. Inflamed IFP secretes various inflammatory cytokines and adipokines, such as IL-6 and adiponectin, that contribute to inflammation and cartilage degradation [6]. It also synthesizes chemotactic factors such as osteopontin, which has been associated with OA progression [7].

As is well documented, running is widely recognized as an excellent form of exercise that promotes overall health and well-being [8]. However, its impact on OA is intensity-dependent. Previous studies have established that low to medium-intensity running is beneficial for joint health [9]. In contrast, high-intensity running generates supra-physiological joint loading, which leads to tissue damage and degeneration [10]. However, previous studies have primarily investigated the effects of running on cartilage and subchondral bone, while research regarding the IFP remains scarce. To date, only one study has examined the effects of different running intensities on the IFP [11], reporting that high-intensity running induced IFP fibrosis, whereas low and medium-intensity running elicited no significant alterations. Furthermore, given that this study was conducted in healthy joints, the effects of different running intensities on the IFP in the context of OA pathology remain elusive. Consequently, the present study aimed to investigate the effects of different running intensities on IFP remodeling in a rat model of OA and explore the association between exercise-induced IFP remodeling and subsequent changes of OA symptoms, cartilage degradation, and subchondral bone damage.

## Materials and methods

### Experimental animals

Wistar rats were purchased from SPF Biotechnology Co., Ltd. (Beijing, China). The animal experimental protocols were approved by the Animal Ethics Committee of the Tenth Affiliated Hospital of Southern Medical University (Dongguan People’s Hospital) (Approval number: IACUC-AWEC-202508009). ARRIVE guidelines were followed. To avoid gender bias, thirty 8-week-old male Wistar rats were housed under specific pathogen-free (SPF) conditions with controlled environmental parameters, with a 12-hour light/dark cycle and an ambient temperature of 22 ± 1 °C. All cages were randomly positioned and animals were allowed to move freely within the cages and had ad libitum access to food and water.

### Study design and exercise protocols

**Fig. 1:**
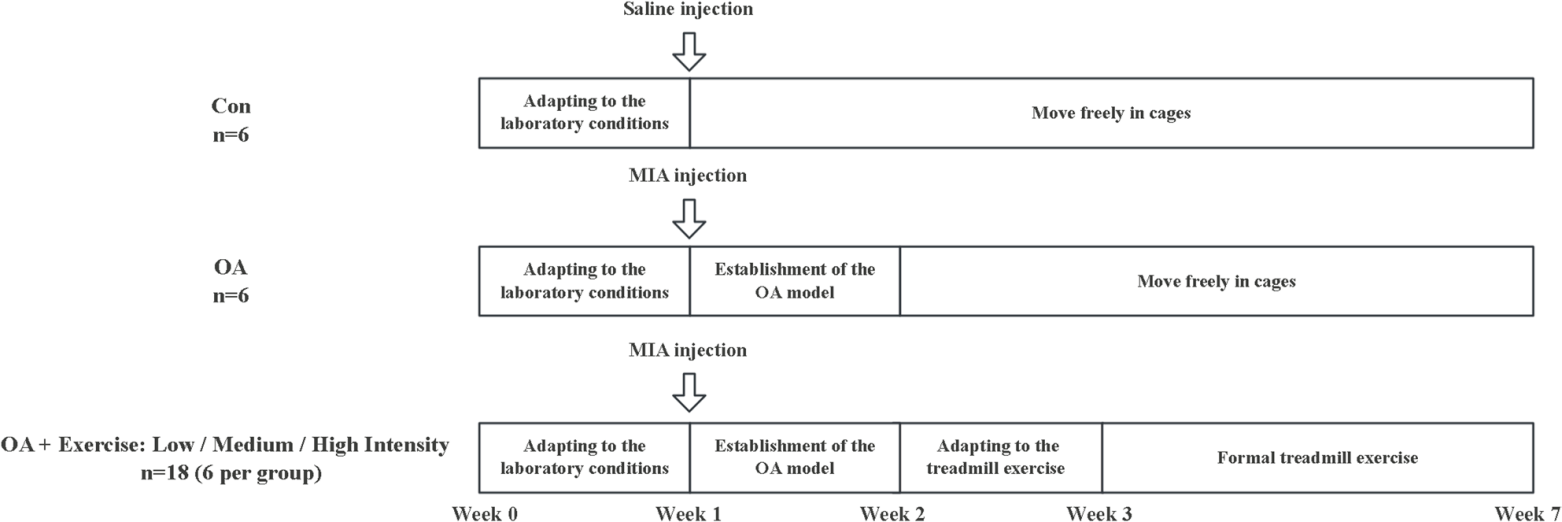
Experimental timeline. A total of 30 Wistar rats were divided into five groups: a control group, an OA group, an OA with low-intensity running (OAL) group, an OA with medium-intensity running (OAM) group, and an OA with high-intensity running (OAH) group. All groups underwent environmental adaptation during weeks 0–1. The Con group received an intra-articular saline injection followed by cage activity, whereas the OA and OA+Exercise groups received an MIA injection followed by a one-week period to establish the OA model. Subsequently, the OA+Exercise group completed a 1-week treadmill adaptation prior to a standardized 4-week treadmill training protocol, whereas the Con and OA groups maintained unrestricted cage activity throughout the intervention period

The experimental protocol is schematically illustrated in Fig. 1. Refering to a previous study [11], rats were randomly assigned to five groups via the lottery method (*n* = 6/group): a control group, an OA group, an OA with low-intensity running (OAL) group, an OA with medium-intensity running (OAM) group, and an OA with high-intensity running (OAH) group. Under 2% isoflurane anesthesia, OA was induced in OA and OA + running groups via intra-articular injection of 50 µL MIA (20 mg/mL in saline), while the control group was administered 50 µL saline. After MIA injection, rats were allowed a 7-day recovery period to ensure the establishment of the OA model and resolution of the acute inflammatory phase [12]. Subsequently, rats in the OA + running groups underwent 1-week treadmill acclimation training (10 m/min, 10 min/day, 5 days/week), followed by a 4-week formal training regimen. The OAL, OAM, and OAH groups ran at speeds of 12 m/min, 16 m/min, and 21 m/min, respectively, for 30 min per day, 5 days per week; these speed parameters were based on a previous study [9]. Rats in the control and OA groups were allowed to move freely in their cages. All animals were euthanized within 48 h after the final exercise session for tissue collection.

### Histological assessment

Samples were meticulously dissected and fixed in 4% paraformaldehyde for 24 h, followed by decalcification in 10% ethylenediaminetetraacetic acid (EDTA) solution for 4 weeks. After dehydration through a graded ethanol series, the specimens were embedded in paraffin and sectioned at a thickness of 5 μm for histological analysis. Next, the sections were stained with picro-sirius red [13], hematoxylin-eosin (HE), and Safranin O-Fast Green [14] according to standard protocols. The picro-sirius red-stained sections were observed under polarized-light microscopy to visualize collagen fibers, and the percentage area of fibrosis was quantified using ImageJ software. Additionally, fibrosis and inflammation in the IFP were semiquantitatively scored based on established criteria [15]. Cartilage damage was evaluated using the Osteoarthritis Research Society International (OARSI) grading system (0–6) and the Mankin scoring system (0–14 points) [16]. All semi-quantitative scoring and analyses were performed by an investigator (Zh.H) blinded to sample group assignments.

### Immunohistochemical assessment

After deparaffinization and rehydration, antigen retrieval was performed using microwave-heated sodium citrate antigen repair solution for 15 min. Endogenous peroxidase was blocked with 3% hydrogen peroxide for 10 min, and nonspecific binding was minimized by incubating sections with 10% goat serum for 30 min. The sections were then incubated overnight at 4℃ with the following primary antibodies: anti-Arg-1 (16001-1-AP, 1:100, Proteintech), anti-iNOS (22226-1-AP, 1:800, Proteintech), anti-Col I (66761-1-IG, 1:500, Proteintech), and anti-α-SMA (14395-1-AP, 1:1000, Proteintech). After washing with PBS, the sections were incubated with a secondary antibody for 1 h at room temperature. Subsequently, color development was performed using diaminobenzidine, followed by counterstaining with hematoxylin. Finally, the sections were dehydrated and sealed. All image analyses were performed by an investigator (Zh.H) blinded to sample group assignments.

### Micro-CT analysis

Micro-CT imaging of the knee joints was performed using a Quantum GX2 system (PerkinElmer, Shelton, Connecticut, USA) with the following parameters [17]: tube voltage 70 kV, current 114 µA, scan time 4 min, and voxel size 72 μm. The region of interest was defined as the subchondral trabecular bone region extending 1 mm below the growth plate in the tibial epiphysis. Three-dimensional reconstruction and analysis of bone microarchitecture parameters were performed using the Analyze 14.0 software (AnalyzeDirect, Overland Park, Kansas, USA). All quantitative scoring and analyses were performed by an investigator (Zh.H) blinded to sample group assignments.

### Assessment of pain-avoidance behavior

In rats, asymmetry in weight-bearing between the bilateral hind limbs is recognized as a behavioral indicator of musculoskeletal pain [18]. In the present study, weight-bearing asymmetry was quantified using an incapacitance meter (Kew Instrumentation, kw-11 A) [19]. Briefly, animals were placed in a testing chamber with their ipsilateral and contralateral hind limbs positioned on separate force transducer panels and allowed to acclimatize. After stabilization, the average force on each panel was recorded over a 5-second period. The investigator was blinded to the weights measured in the test. Weight-bearing asymmetry was calculated as (ipsilateral/contralateral) × 100%. A value of 100% indicated equal weight distribution and absence of significant pain. Measurements were performed at weeks 0, 1, 2, 3, 4, 5, and 6 after intra-articular injection of MIA or saline.

### Statistical analysis

Statistical analyses were performed using GraphPad Prism (version 9.5.1) and R software (version 4.5.1). All data were presented as the mean ± standard deviation. Comparisons of multi-group trends over time (including body weight and load asymmetry) were analyzed using repeated-measures two-way analysis of variance (ANOVA), with the Greenhouse-Geisser correction applied. Post-hoc multiple comparisons were conducted using the Holm-Sidak method. For multigroup comparisons, either ordinary one-way ANOVA or Welch’s ANOVA was employed, based on the result of the Brown-Forsythe test for homogeneity of variances. Accordingly, post hoc multiple comparisons were performed using either Tukey’s test or Dunnett’s T3 test. Correlations were assessed using Spearman’s correlation analysis. A P value of less than 0.05 was considered statistically significant.

## Results

### Body weight

**Fig. 2:**
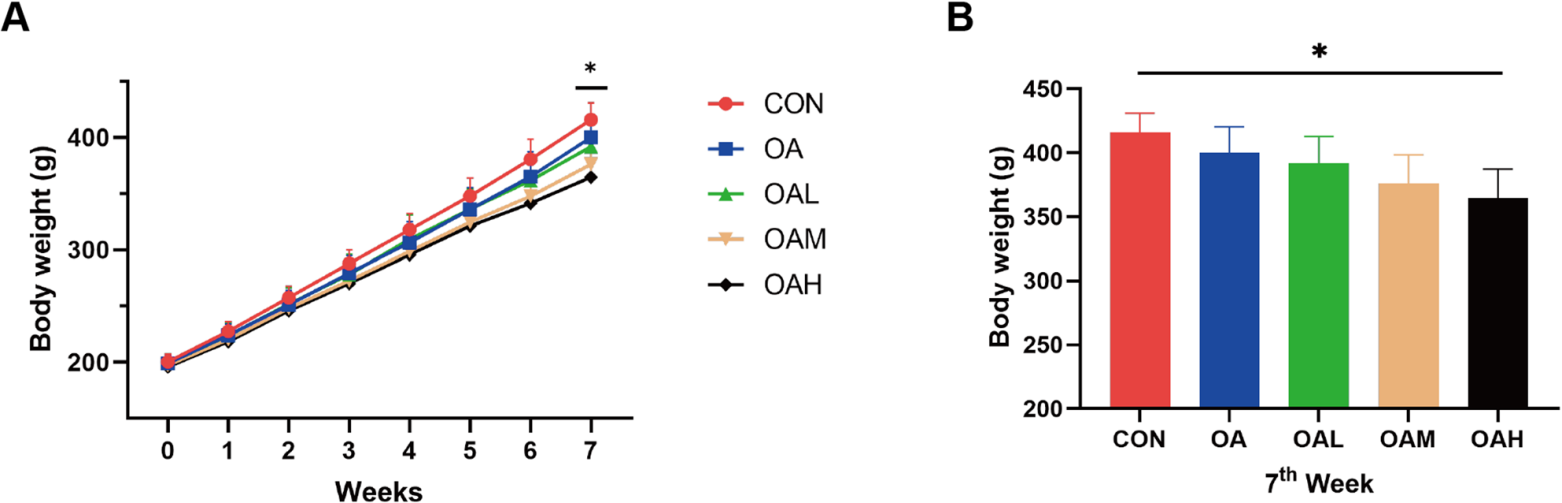
Temporal changes in body weight across experimental groups. (**A**) Weekly body weight measurements (g) of Wistar rats from Weeks 0 to 7. (**B**) Body weight at week 7 in each group. *P < 0.05

As displayed in Fig. 2, body weight was measured weekly throughout the experimental period. While all groups exhibited a progressive increase in body weight, significant differences between groups were noted at week 7 (Fig. 2A). Specifically, the OAH group exhibited a significantly lower body weight compared to the CON group at this time point (*p* = 0.002). In contrast, no significant differences were observed among any other groups (Fig. 2B).

### Differential effects of running intensity on IFP remodeling

**Fig. 3:**
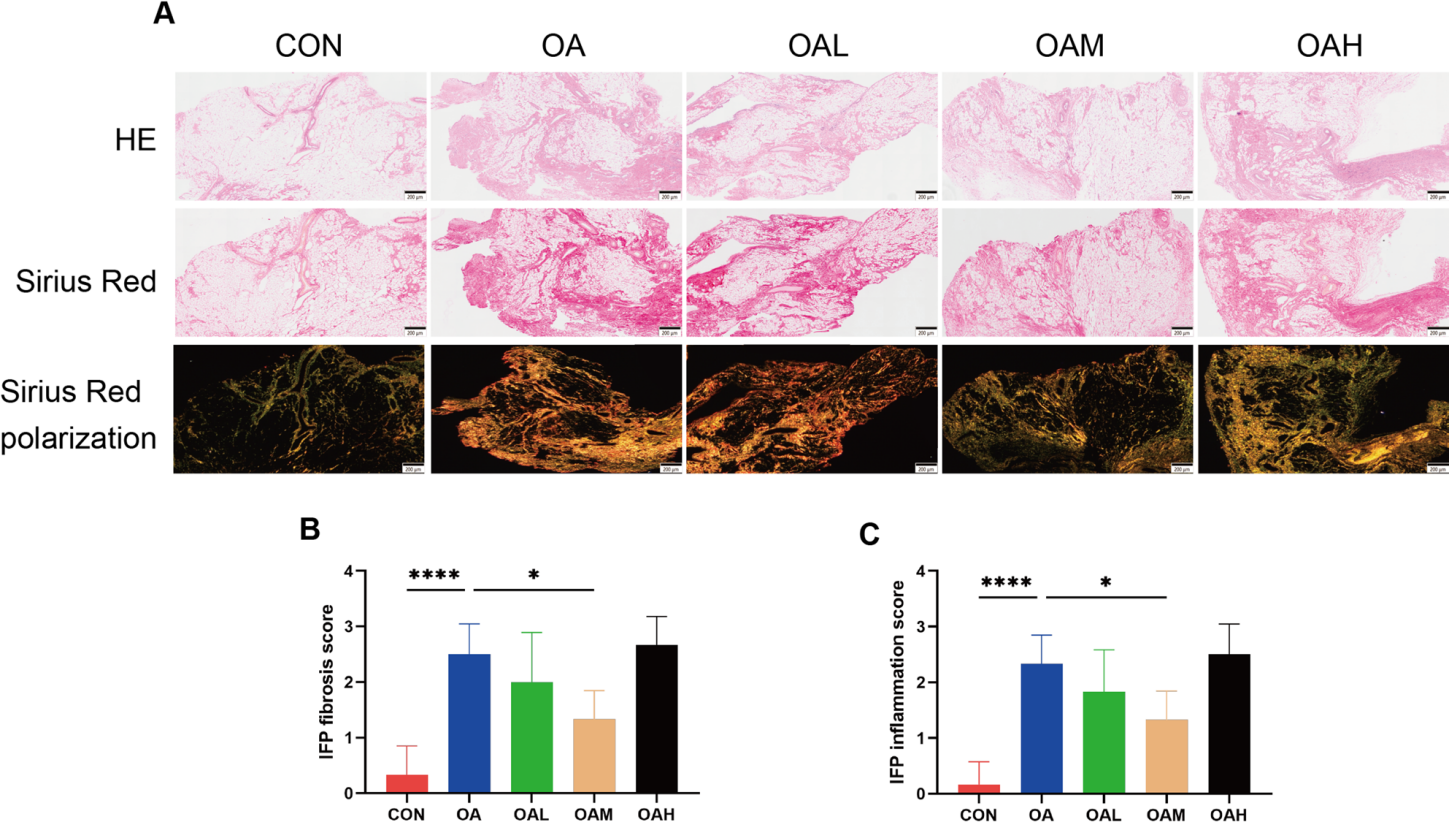
Histological features of the IFP across groups. (**A**) Representative images of IFP sections stained with HE, Sirius Red, and Sirius Red under polarized light microscopy. Scale bars = 200 μm. (**B**) Semiquantitative evaluation of IFP fibrosis. (**C**) Semiquantitative evaluation of IFP inflammation. Data are presented as mean ± SD. *P < 0.05, ****P < 0.0001

Histological analysis was performed to evaluate the effects of different running intensities on IFP remodeling. As depicted in Fig. 3A, HE staining was employed to assess cellularity and inflammatory changes, while picrosirius red staining under polarized light was applied to quantify collagen deposition and fibrosis. As anticipated, polarized imaging revealed distinct collagen fiber patterns among the groups. The CON group had minimal collagen deposition, while the OA group showed extensive collagen accumulation. Among the exercise groups, the OAM group showed a significantly lower degree of fibrosis compared to the OA group (*p* = 0.023). No significant differences were observed between OA and OAL group. The degree of fibrosis was comparable between the OAH group and the OA group (Fig. 3B). In the HE-stained images, the OA group showed significantly higher inflammation score than the CON group. Among the exercise groups, only the OAM group showed a significantly lower inflammation score compared with the OA group (*p* = 0.035). The OAH group exhibited inflammatory changes similar to the OA group (Fig. 3C).

**Fig. 4:**
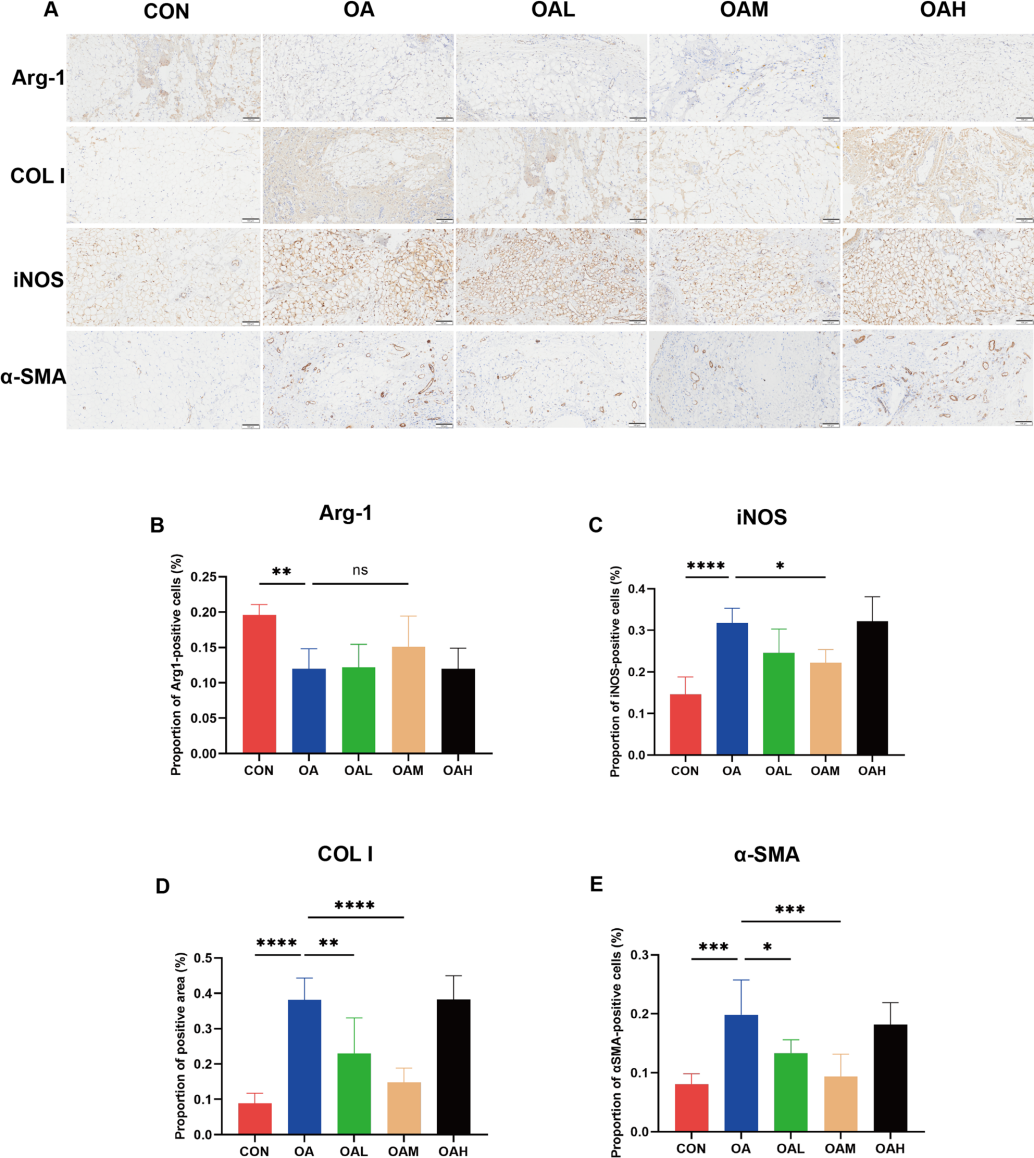
IHC analysis of macrophage polarization and fibrosis-related markers in IFP across groups. (**A**) Representative IHC staining images of Arg-1, COL I, iNOS, and α-SMA expression in IFP. Scale bars = 100 μm. (**B**) Semi-quantitative analysis of the proportion of Arg-1-positive cells. (**C**) Semi-quantitative analysis of the proportion of iNOS-positive cells. (**D**) Semi-quantitative analysis of the proportion of COL I-positive area. (**E**) Semi-quantitative analysis of the proportion of α-SMA-positive cells. Data are presented as mean ± SD. *P < 0.05, **P < 0.01, ***P < 0.001, ****P < 0.0001, ns (not significant)

To investigate the molecular alterations underlying IFP remodeling induced by different running intensities, IHC was performed (Fig. 4) to detect markers of macrophage polarization (Arg-1 and iNOS) and fibrosis (COL I and α-SMA). Regarding macrophage polarization (Fig. 4A–C), Arg-1 expression levels were highest in the CON group and significantly decreased in the OA group (*P* = 0.002), with no significant differences observed among the exercise intervention groups. In contrast, iNOS expression levels were minimal in the CON group but markedly increased in the OA group (*P* < 0.0001). Among the exercise groups, only the OAM group showed a significant reduction in iNOS expression levels compared to the OA group (*P* = 0.011). Concerning fibrosis markers (Fig. 4D and E), COL I and α-SMA were weakly expressed in the CON group but markedly upregulated in the OA group (*P* < 0.0001 for COL I, *P* < 0.001 for α-SMA). Compared to the OA group, the OAL and OAM groups showed significantly lower expression levels of COL I and α-SMA, especially in the OAM group (*P* < 0.001).

### Differential effects of running intensity on cartilage and subchondral bone

**Fig. 5:**
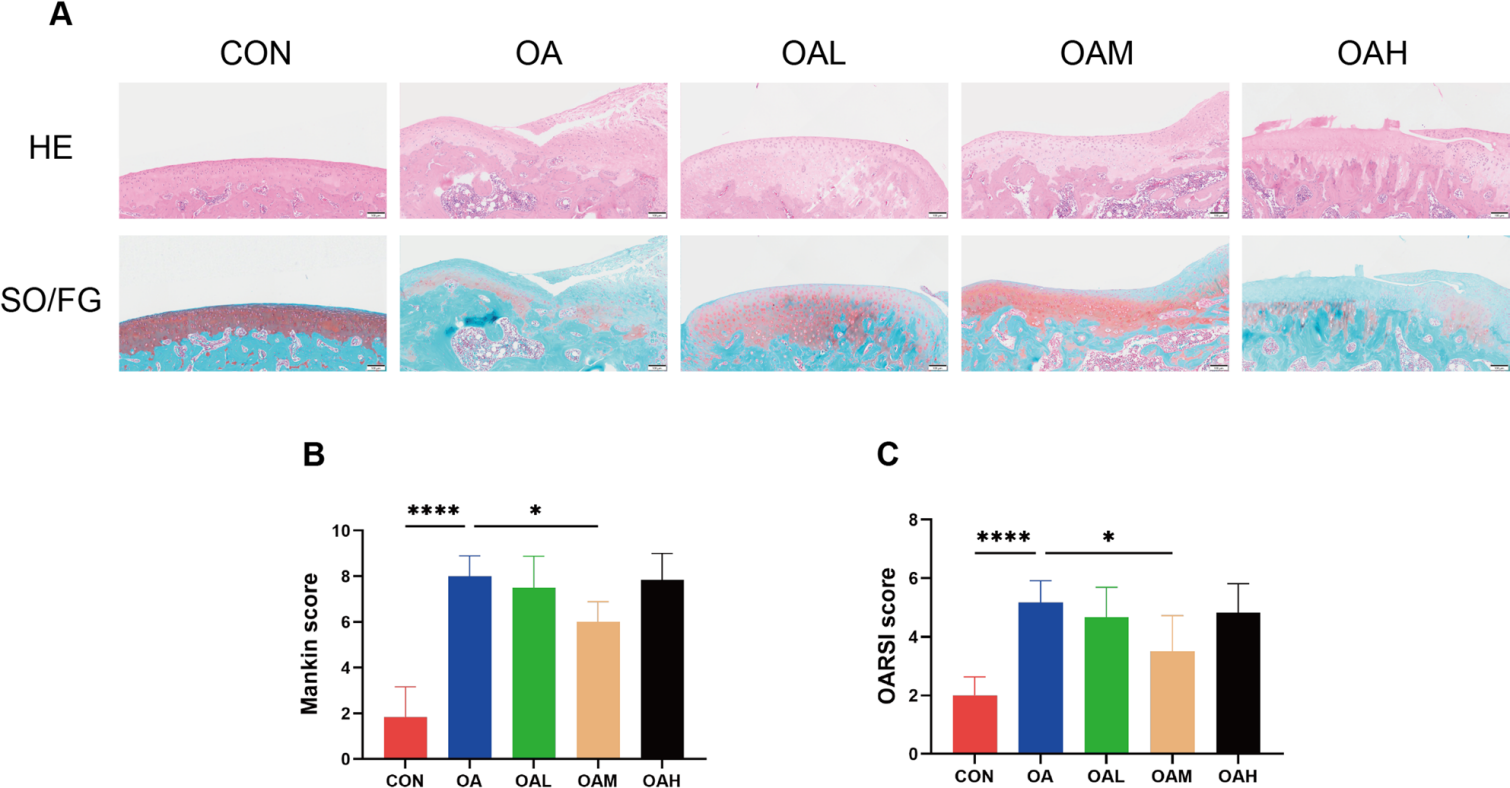
Histological features of the cartilage across groups. (**A**) Representative images of cartilage stained with HE and SO/FG. Scale bars = 100 μm. (**B**) Semi-quantitative analysis of the Mankin score. (**C**) Semi-quantitative analysis of OARSI score. Data are presented as mean ± SD. *P < 0.05, ****P < 0.0001

To evaluate the effects of different running intensities on cartilage, histological analysis was performed with HE and Safranin O/Fast Green staining to assess cartilage integrity. As delineated in Fig. 5A, the CON group exhibited intact cartilage structure, while the OA group showed evidence of surface fissures, chondrocyte clustering, and marked loss of glycosaminoglycans. In contrast, the OAM group showed significant improvement in these pathological changes compared to the OA group. The OAL group also showed partial improvement, while the OAH group exhibited morphological features similar to the OA group. Semi-quantitative analysis unveiled that the OAM group had significantly lower Mankin and OARSI scores compared to the OA group (*P* = 0.042 for Mankin, *P* = 0.04 for OARSI). The OAL and OAH groups did not show significant differences compared with the OA group (Fig. 5B and C).

**Fig. 6:**
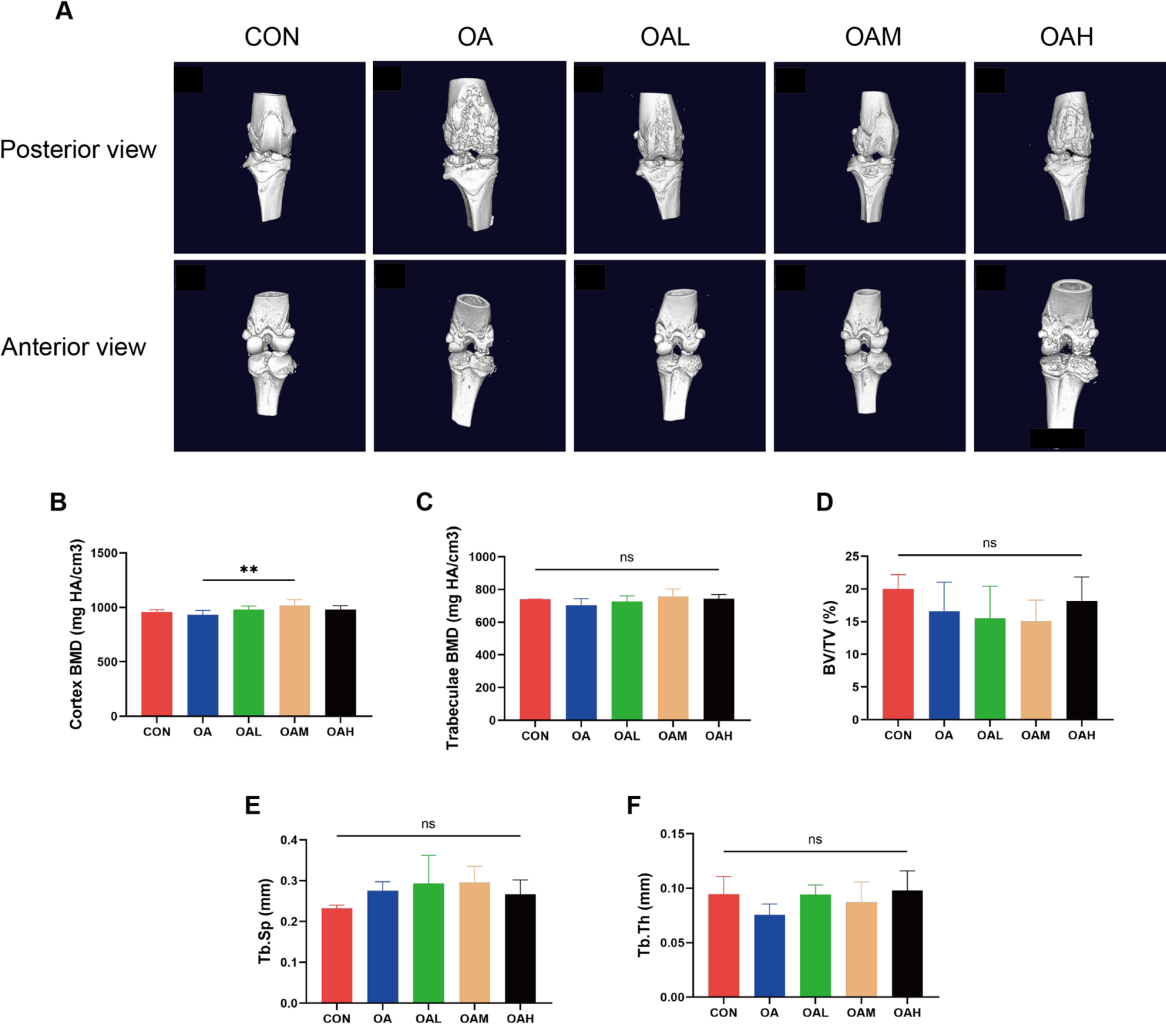
Subchondral bone microarchitecture across groups. (**A**) Representative 3D images of the knee joint in posterior and anterior views. (**B**) Analysis of cortex BMD (mg HA/cm³). (**C**) Analysis of trabeculae BMD (mg HA/cm³). (**D**) Analysis of BV/TV (%). (**E**) Analysis of Tb.Sp (mm). (**F**) Analysis of Tb.Th (mm). Data are presented as mean ± SD. **P < 0.01, ns (not significant)

Micro-CT analysis was performed to investigate the effects of different running intensities on subchondral bone microstructure. As shown in Fig. 6A, three-dimensional reconstruction images (posterior and anterior views) uncovered that the CON group exhibited intact subchondral bone surfaces without evidence of damage or osteophyte formation. On the other hand, the OA group exhibited significant bone erosion and osteophyte formation. Among the exercise groups, the OAL and OAM groups showed improvement in bone morphology compared to the OA group, while the OAH group remained comparable to those observed in the OA condition. Quantitative analysis of subchondral bone parameters (Fig. 6B–F) indicated that only cortex BMD showed significant differences. Specifically, the OAM group had significantly higher cortex BMD than the OA group (*P* = 0.008). Other parameters, including trabeculae BMD, BV/TV, Tb.Sp, and Tb.Th were comparable across groups.

### Differential effects of running intensity on pain in the affected limb

**Fig. 7:**
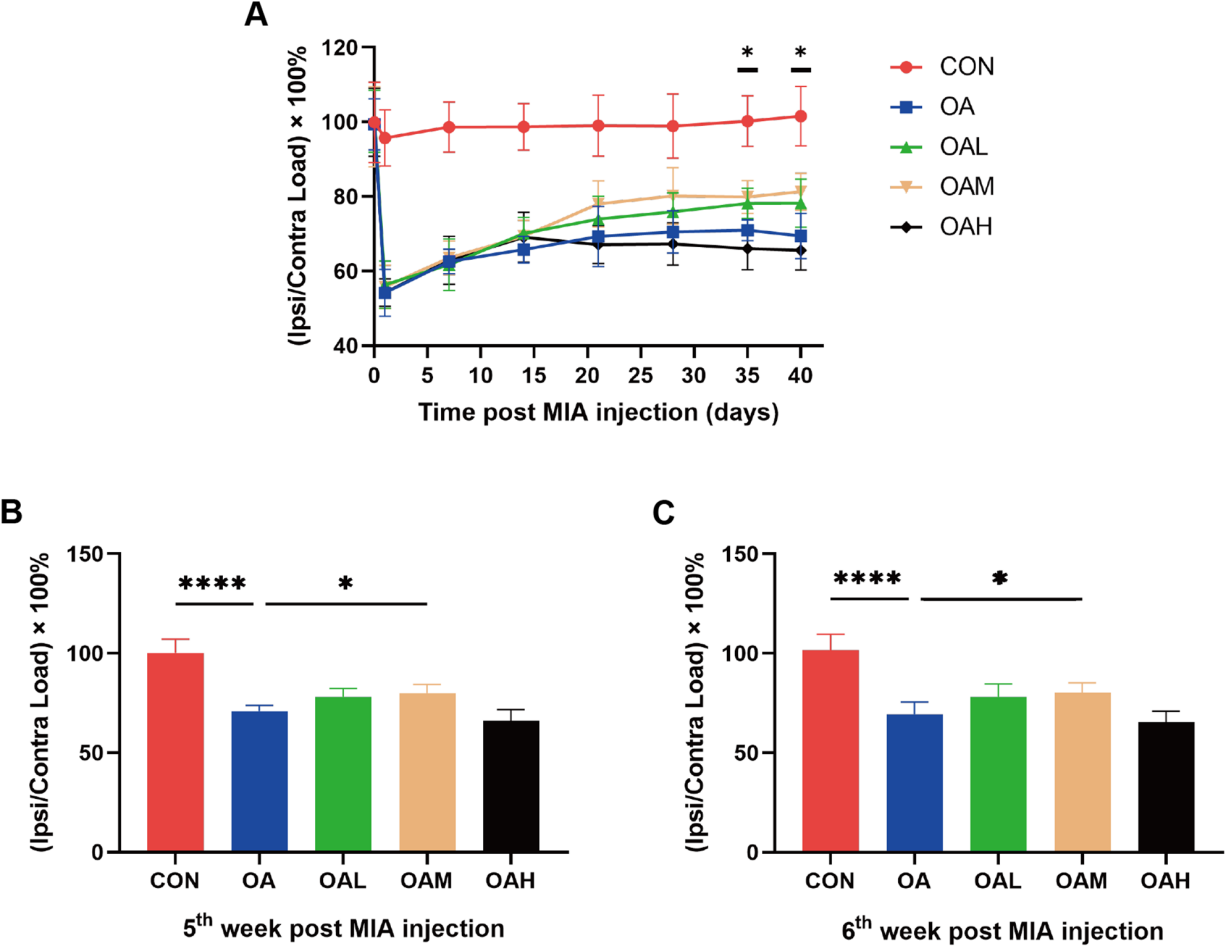
Ipsilateral limb weight-bearing ratio across experimental groups. (**A**) Temporal changes in ipsilateral limb weight-bearing ratio post MIA injection. (**B**) Analysis of ipsilateral limb weight-bearing ratio at the 5th week post MIA injection. (**C**) Analysis of ipsilateral limb weight-bearing ratio at the 6th week post MIA injection. Data are presented as mean ± SD. *P < 0.05, ****P < 0.0001

To evaluate temporal changes in weight-bearing of the affected limb following MIA injection, weight distribution was measured over time. As presented in Fig. 7A, the CON group maintained a stable weight-bearing ratio at approximately 100% throughout the observation period, indicative of no significant pain. Conversely, the OA and exercise groups exhibited a significant decrease in weight-bearing on day 1 post-MIA injection, followed by a gradual recovery that plateaued around week 3. No significant intergroup differences were observed among all exercise groups and the OA group before week 5. However, significant intergroup differences emerged at weeks 5 and 6 post-injection (Fig. 7B, C). Indeed, the OAM group had a significantly higher weight-bearing ratio than the OA group (*P* = 0.033 for week 5, *P* = 0.042 for week 6), indicating a significant reduction in pain. However, no significant differences were noted between the remaining exercise groups and the OA group.

### Exercise-induced IFP alteration is associated with changes in pain and cartilage-subchondral bone integrity

**Fig. 8:**
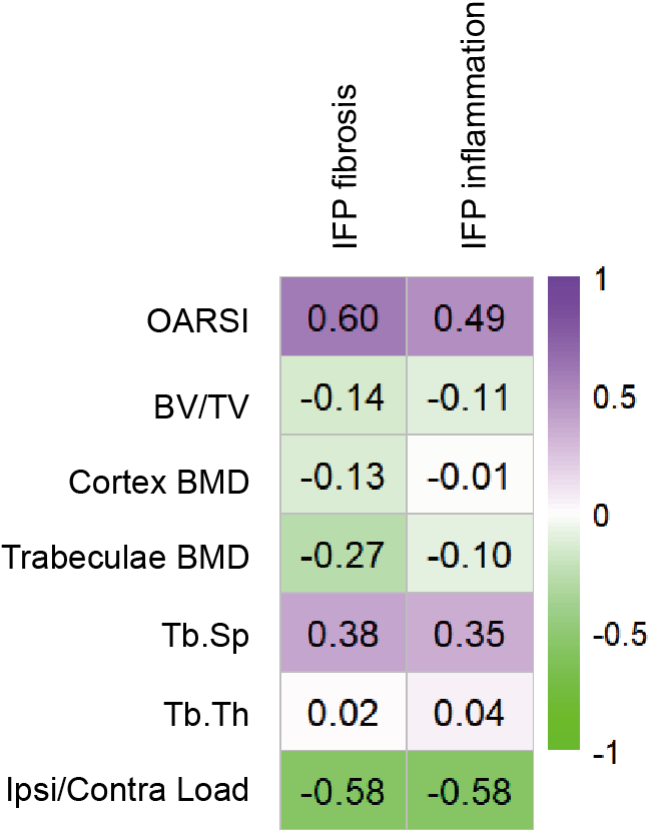
Heatmap of correlation between IFP remodeling and structural damge and pain severity. Colors in the heatmap correspond to the strength and direction of correlation, where purple means a positive correlation, green means a negative correlation. Values in the heatmap boxes represent the correlation coefficient

To explore the association between IFP remodeling, cartilage damage, subchondral bone parameters, and ipsilateral limb pain, a correlation heatmap was established. As shown in Fig. 8, OARSI scores were significantly positively correlated with IFP fibrosis (*r* = 0.60) and inflammation (*r* = 0.49), signifying that higher levels of IFP fibrosis and inflammation were associated with more severe cartilage damage. Among subchondral bone parameters, Tb.Sp showed a moderate positive correlation with IFP fibrosis (*r* = 0.38) and IFP inflammation (*r* = 0.35), suggesting that more severe IFP remodeling was linked to increased trabecular separation. In contrast, BV/TV, cortex BMD, Tb.Th, and trabeculae BMD showed weak correlations with IFP remodeling. Notably, ipsilateral weight-bearing was strongly negatively correlated with IFP fibrosis and inflammation (*r* = -0.58 for each), indicating that more severe IFP remodeling was associated with greater pain severity.

## Discussion

This study investigated the intensity-dependent effects of treadmill running on IFP remodeling in osteoarthritic rats. Moderate-intensity running conferred significant benefits, including reduced IFP fibrosis and inflammation, improved cartilage integrity, increased subchondral cortical bone density, and alleviated pain. In contrast, low-intensity and high-intensity running did not show protective effects, without exacerbating joint damage compared with the OA group. Furthermore, correlation analysis signaled that improvements in IFP remodeling were significantly associated with pain relief and joint preservation. Collectively, these findings suggest that the effect of running on IFP remodeling is intensity-dependent and indicate a potential link between IFP and exercise-induced joint protection.

These findings demonstrate that moderate-intensity running ameliorates IFP remodeling, consistent with the findings of previous studies. Zeng et al. compared the effects of different running intensities on IFP remodeling in healthy rats and reported that moderate-intensity running assisted in maintaining IFP homeostasis [11], whilst another study using voluntary wheel running also supported its beneficial role in IFP homeostasis [20]. The present study extends these findings to a pathological OA context, demonstrating that moderate-intensity running can reverse OA-induced pathological IFP remodeling. The underlying mechanisms are likely multifactorial. To begin, appropriate mechanical stimulation may improve the intra-articular microenvironment, thereby ameliorating IFP remodeling. This notion is supported by previous research showing that moderate-intensity running can mitigate synovial macrophage M1 polarization through optimal mechanical strain [21]. Secondly, exercise can enhance IFP microcirculation to maintain tissue homeostasis. In agreement with this, isometric quadriceps exercise has been documented to improve IFP perfusion [22]. Although the exercise modalities differ, this mechanism may partially account for the effects noted in this study. Thirdly, exercise-induced exerkines such as irisin may modulate IFP remodeling. Previous studies have demonstrated the beneficial effects of exercise-derived exerkines on knee joint structures such as cartilage and subchondral bone [23]. However, few studies have explored whether exerkines target the IFP or their effects on it. Given that the IFP is primarily composed of adipose tissue, which is an established downstream target of exerkines [24], we hypothesize that the IFP may also serve as a key target of exerkines, and this could be a focus of future research.

Importantly, the findings of this study indicate that high-intensity running did not confer significant protective effects on IFP remodeling in OA rats; however, it did not exacerbate the pathology. In contrast, Zeng et al. reported that high-intensity running induces IFP fibrosis in healthy rats [11]. This discrepancy may be attributed to two primary factors. Firstly, the animal models differed. This study used OA rats with pre-existing severe IFP pathology, in which additional damage induced by high-intensity running might have been masked. Secondly, the exercise protocols differed. Zeng et al. used a more strenuous regimen (26.8 m/min, 10° incline, 60 min), which could elicit more severe tissue damage, whereas the high-intensity protocol used in this study was less aggressive. Theoretically, excessive mechanical load from high-intensity running may promote the M1 polarization of macrophages, triggering the release of pro-inflammatory mediators and initiating intra-articular inflammation [14]. Repetitive inflammatory stimulation can subsequently lead to IFP fibrosis [25]. In the present study, the high-intensity running parameters applied were relatively moderate and did not induce significant additional tissue damage. Future investigations could employ more intensive exercise regimens to further explore this relationship.

It is worthwhile emphasizing that a significant positive correlation was identified between the degree of IFP fibrosis/inflammation and pain severity. The OAM group, which showed the greatest improvement in IFP remodeling, also exhibited the most significant pain relief, strongly implicating IFP as a critical link connecting exercise intervention to analgesia. The mechanism may be attributed to the IFP’s rich innervation [26]; pathological remodeling can directly stimulate sensory nerve endings and generate pain [27]. Exercise, by reducing IFP fibrosis-induced stiffness and local inflammation, may decrease both mechanical and chemical stimulation of these nerves, thereby alleviating pain. Besides, improved IFP function might indirectly reduce mechanical stimulation on other pain-sensitive structures, such as the synovium [28] and subchondral bone [29], by optimizing mechanical distribution within the joint.

Correlation analysis revealed a positive association between IFP pathological changes and cartilage damage. The OAM group demonstrated the most substantial improvements in both IFP remodeling and cartilage integrity, implying that exercise may indirectly influence cartilage integrity by modulating the IFP. On one hand, a healthier, more compliant IFP can more effectively perform its biomechanical cushioning function, thereby limiting abnormal stress transmitted to the cartilage [2]. On the other hand, a non-inflammatory IFP secretes fewer pro-inflammatory and pro-catabolic factors such as IL-6 [30] and osteopontin [7], thereby mitigating catabolic effects on cartilage. The cartilage improvement observed in the OAM group may be partly ascribed to the appropriate mechanical and biochemical environment resulting from improved IFP remodeling.

The differential response of subchondral bone parameters to exercise intervention suggests a compartment-specific adaptation pattern. While cortex BMD showed significant intergroup differences, trabecular parameters, including trabecular BMD, BV/TV, Tb.Th, and Tb.Sp did not show significant differences across groups. This selective response may be attributed to the distinct biological characteristics of bone compartments. Cortical bone, as the primary load-bearing structure, demonstrates greater responsiveness to mechanical stimulation through enhanced mineralization [31]. In comparison, the trabecular network may require longer intervention periods to manifest detectable structural adaptations, consistent with existing findings that substantial trabecular remodeling typically occurs in advanced OA [32]. The limited sample size may also be responsible for the lack of statistical significance. The significant correlation between Tb.Sp and IFP pathology reflects the pathologically driven structural remodeling of trabecular bone. The inflammatory microenvironment induced by IFP remodeling is likely a contributor to trabecular bone resorption [27]. Notably, Tb.Sp appears to be more sensitive than other parameters in capturing early alterations in trabecular architecture [33].

This study holds several clinical implications. Firstly, the results highlight the need for precise intensity control when designing exercise regimens for OA patients. Moderate intensity appears to be a critical window for exerting joint-protective effects. This observation offers direct guidance for rehabilitation medicine. For patients with knee OA, they may undergo maximal oxygen uptake (VO₂max) testing prior to running, and then adjust running speed to 40–60% of their VO₂max based on individual physical condition. Secondly, the strong correlations between IFP remodeling and both pain perception and structural joint damage suggest that IFP characteristics could serve as an objective indicator of disease status. In the clinical setting, non-invasive imaging modalities such as MRI and ultrasound could be used to monitor IFP changes, providing a valuable approach for disease monitoring and treatment evaluation. The parallel improvements in IFP remodeling and clinical symptoms in the OAM group further suggest that IFP status may aid in predicting individual response to exercise intervention, potentially guiding personalized treatment strategies.

The strengths of this study include the integrated use of histological, immunohistochemical, micro-CT, and behavioral analyses to comprehensively demonstrate the differential effects of running intensity on the IFP and to establish the association between IFP remodeling and overall joint health. However, some limitations should be acknowledged. To begin, the conclusions are based on an MIA-induced OA model, which primarily reflects the inflammatory phenotype of OA but does not reflect chronic degenerative characteristics, requiring future validation in human studies. Secondly, although significant changes in the IFP structure and function were observed, the upstream and downstream mechanisms were not systematically explored. Additionally, due to animal welfare considerations, the sample size was 6 per group, which represents the minimum sample size required for statistical analysis. From the perspective of statistical power, this sample size is relatively insufficient. Future studies could appropriately increase animal numbers in compliance with the 3R principles to enhance the robustness and generalizability of the results. Finally, the study did not conduct rescue experiments to directly establish a causal relationship between IFP modulation and the therapeutic benefits of exercise, which represents a key direction for future investigations.

## Conclusion

In conclusion, treadmill running exerts intensity-dependent effects on IFP remodeling in OA rats, with moderate intensity providing optimal therapeutic benefits. IFP remodeling is significantly associated with joint structural integrity and pain, indicating a potential link between IFP and exercise-induced joint protection.

## Data Availability

Data may be shared by the corresponding author upon reasonable request.
